# Programmatic mapping and estimating the population size of female sex workers, men who have sex with men, people who inject drugs and transgender populations in Kenya

**DOI:** 10.12688/gatesopenres.13623.2

**Published:** 2023-08-18

**Authors:** Janet Musimbi, Helgar Musyoki, Mary Mugambi, Shem Kaosa, Japheth Kioko, Diane Aluko, Waruiru Wanjiru, Solomon Wambua, Ravi Prakash, Shajy Isac, Parinita Bhattacharjee, Faran Emmanuel

**Affiliations:** 1 Partners for Health and Development in Africa, Nairobi, 00506, Kenya; 2 The Global Fund, 1218 Grand-Saconnex, Geneva, Switzerland; 3 National AIDS and STI Control Programme, Ministry of Health, Ministry of Health, Nairobi, 00202, Kenya; 4 Global Health Program, University of California San Francisco, Nairobi, 00400, Kenya; 5 Key Population Consortium of Kenya, Nairobi, 00100, Kenya; 6 Institute for Global Public Health, University of Manitoba, Winnipeg, Manitoba, R3E 0T6, Canada

**Keywords:** Key Populations, programmatic mapping, population size estimates

## Abstract

Introduction: Effective coverage of Human Immunodeficiency Virus prevention services for Key Populations (KPs) including female sex workers (FSWs), men who have sex with men (MSM), people who inject drugs (PWID) and transgender (TG) people necessitates periodic validation of physical venues and size estimates. Kenya conducted a robust size estimation of KPs in 2012 and a repeat mapping and size estimation exercise was conducted in 2018 to update KP Size Estimates and sub-typologies within each County for calculation of realistic program indicators.

Methods: A prospective mixed methods programmatic mapping approach adopted comprised two steps. The first step involved consolidating and documenting all known venues where KPs congregate while the second step included visiting and validating these venues confirming their active status. Data were collected in 34 out of 47 Counties in Kenya between January and March 2018. Data collected included estimated number of KPs (range), venue typology and timing of operation of each venue.

Results: We estimated a total number of 167,940 (129,271 to 206,609) FSWs; 32,580 (24,704 to 40,455) MSM; 16,063 (12,426 to 19,691) PWIDs; 10,951 (8,160 to 13,742) and 4,305 (2,826 to 5,783) transgender people congregating at 10,250, 1,729, 401 and 1,218 venues respectively. Majority of the venues for FSW (81%), MSM (64%) and transgender people (67%) were bars with and without lodging, PWIDs were mostly found on streets and injecting dens (70%). Around 9% of FSW and MSM and11% of PWIDs were below the age of 18 years.

Conclusion: This study provided information on young KPs, female PWIDs, MSWs and for the first time, TG people in Kenya. The exercise updated size estimates of KPs by typology and provided new evidence for resource allocation, planning of interventions and targets. Programmatic mapping continues to be a useful approach supporting programs to achieve high levels of coverage and prioritize resources.

## Introduction

Key populations (KPs) are disproportionately affected by human immunodeficiency virus (HIV) compared to the general population ^1, 2^. In 2018, 54% of all new adult HIV infections globally were among KPs (including sex workers, people who inject drugs, prisoners, transgender people, and men who have sex with men) and their sexual partners indicating their elevated risk of HIV infection ^3^. This elevated risk of HIV among KPs and their sexual partners is attributed to risky sexual behaviors such as multiple sexual partners, unprotected sex and sharing contaminated needles but also in part to stigma and discrimination, high violence and barriers to HIV prevention, care, treatment, and other services among these populations ^4^. To scale up HIV prevention interventions tailored to local needs, it is important to understand the epidemic and its drivers as well as its geographic localization, then develop a public health response using these local updated data ^5, 6^. Programmatic mapping and size estimation serves as a critical step to develop and scale-up HIV prevention programs for KPs. It helps to estimate population size, understand where these populations are located and how to reach them for an effective program response, provides evidence to decision makers on numbers within the counties and therefore supports in resource mobilization for interventions for KPs. It also provides denominators which can be used to track the progress of a program and interventions ^7, 8^. A review conducted in Africa showed that less than half of the 54 countries in Africa had estimated the size of KPs and published these data ^9^. Within Eastern Africa, Tanzania, Uganda and Kenya were the only countries that have conducted and published KP size estimations ^10– 12^.

Kenya exhibits a mixed HIV epidemic; generalized among the adult population and concentrated within KPs. It is among the ten countries in southern and eastern Africa that account for 80% of all people living with HIV (PLHIV) globally ^13^. HIV prevalence among KPs is high with 18.9% among people who inject drugs (PWID) ^14^, 29.5% among female sex workers (FSW) and 18.2% in men who have sex with men (MSM) ^15^. Recognizing the importance of KPs in the HIV response, the 2014/15-2018/19 Kenya AIDS Strategic Frameworks (KASF) prioritized FSW, MSM, transgender people (TG) and PWIDs in the HIV response to reduce new HIV infections by 75% nationally ^16– 18^. To comprehend the scope of the HIV prevention response, Kenya first conducted programmatic mapping and size estimation of KPs in 2012 where a total of 10,670 FSW hotspots with an estimated number of 103,298 FSWs; 1,585 MSM hotspots with an estimated number of 10,033 MSM and 919 PWID hotspots with an estimated number of 7,850 PWIDs were done. Similarly another size estimation was done in Nairobi where 11,042 MSM, 29,494 FSW and 10,937 PWIDs were estimated ^19, 20^. The results supported the scale up KP interventions in 34 out of 47 counties in the country for example counties that only had programs for the FSW subpopulation, scaled up services to include services for MSM. Since the monitoring and evaluation framework for the KASF 2014/15–2018/19 recommends updating KP size estimates every five years, the Kenya national KP program under the National AIDS and STIs Control Programme (NASCOP) decided to re-map and re-estimate the sizes of KPs in all counties where KP programs were implemented ^17^. Programmatic mapping isn’t designed to identify the total size of KPs, but rather to identify populations that are actively seeking partners at venues (FSW/MSM and TG) and/or congregating for injecting (PWIDs). Kenya for the first time estimated the number of TG people through existing partners who were implementing FSW and MSM programs as there were no programs specifically implementing for this sub-population. This study aimed to update KP size estimates within each County where a KP program was being implemented for the calculation of realistic program targets and indicators, as well as comprehend the changes in KP dynamics over time.

## Methods

### Study design

The study employed a programmatic mapping approach where quantitative data was collected. Programmatic mapping approach maps the sites and spots where key populations engage in risky behavior. It is the systematic identification of locations where KPs congregate and could be reached with services. The term "programmatic" is used to indicate that the mapping is done as part of routine service delivery activities to improve the engagement of KPs with the program and monitor program coverage. It also provides an estimate of the KPs at the venue level and also accounts for the overlap of KPs between different venues. We reviewed and updated the existing hotspots, conducted a physical validation of these spots in addition to identifying new ones. The exercise was led by KP communities, involving existing implementing partners (IPs) and service providers who can also identify existing program and service gaps ^21, 22^. Characteristics and locations of hotspots change, either due to closure or the creation of new hotspots and it is important to validate the existing hotspots and to identify new hotspots. This study revalidated the size estimates of the existing spots and characterized sex work or injecting sites in terms of operational typologies. It also helped identify new hotspots not previously covered by the programme. Virtual hotspots for MSM were not included in the exercise. After consultations with stakeholders and the community it was agreed that the estimates would include 25% of MSM who do not visit physical hotspots and seek partners in the virtual spaces based on a study that mapped the virtual platforms to estimate the population of MSM who use the internet to find sexual partners ^23^.

### Sampling

The size estimates exercice took place in 34 out of the 47 counties in Kenya. We only included counties where HIV prevention programs and services were implemented. Based on this criteria, 34 counties were selected for mapping FSW, 30 counties were selected for mapping MSM, 15 for PWID and 30 counties were selected to map transgender people.

### Data collection

Data were collected between January 2018 to March 2018 by existing IPs in 34 counties (Bomet, Bungoma, Busia, Embu, Homabay, Kajiado, Kakamega, Kericho, Kiambu, Kilifi, Kirinyaga, Kisii, Kisumu, Kitui, Kwale, Laikipia, Nakuru, Machakos, Makueni, Meru, Migori, Mombasa, Muranga, Nairobi, Narok, Nyamira, Nyeri, Siaya, Taita Taveta, Tharaka Nithi, Trans Nzoia, Turkana, Uasin Gishu and Vihiga). This was not a stand alone research study, and was conducted as part of the key population program of the Kenya’s National government initiative. We focused on the counties where HIV prevention programs were already providing services to key populations which were 34 out of the 47 counties. There was a consultative process during the national technical working groups for key populations and it was agreed to conduct the KPSE exercise as part of the programs and to update size estimates of key populations in these 34 counties. At the time of the size estimations only 34 counties had FSW partners implementing for the FSW, 15 counties had PWID implementing partners and 30 counties had MSM implementing partners. These are the counties that were then the focus of the size estimations.

***Level one.*** Data collection was done in two sequential steps: “level one” and “level two”. Level one involved making a comprehensive list of venues and hotspots for different KPs types within each county. This activity was done in collaboration with the peer educators (PEs), outreach workers (ORWs) and program staff from the implementing partners. The exercise involved listing down the known hotspots where KPs congregate and profiling the sites using a form known as level one form (L1 form). The L1 form listed all existing hotspots within a specific sub-location and also the existing hotspot-level information such as hotspot name, location, typology of KPs, overall number of KPs and the details of nearest health facility. This list included those venues and hotspots that were previously mapped by the IPs. The process further involved identifying new venues and hotspots by consulting KP members, peer educators, outreach workers and program staff from IPs.

***Level two.*** Once a complete venue list was developed for each county, the next step known as “level two” involved validating and profiling all known venues or hotspots. In this step, field teams visited each venue or hotspot, identified and interviewed three to five KP members who belong to or operate through that specific venue or hotspot through focus group discussions (FGDs). The FGDs were conducted through structured questions that were part of the form that collected data at level 2 known as Form B. Form B can be found under Extended data ^24^. The interviews took place at the hotspot and were conducted by the PE with support from the ORW and it took about 15 minutes. At the hotspot, a gatekeeper was identified and informed on the process and what information was being collected for informed consent to carry out the exercise. The peer educator for the hotspot then identified either FSWs, MSMs or PWIDs present at the hotpsot to take part in the group discussion. The KP members were identified randomly from the hotspots at the time of the size estimation hence they were found physically at the hotspot during the exercise and were homogeneous in the sense that they belonged to the same KP group. They were all interviewed at the same time and provided the estimate for low, mid and high KPs after discussing among themselves bulding consensus. Data were collected using “level two form” (Form B) which captured hotspot characteristics such as peak day(s), peak time(s), and the approximate numbers of KPs at each site on non-peak days and peak days. In addition, the form captured the numbers of transgender population, number of MSWs, number of PWUD, the numbers of KPs in various age groups, and the numbers of male and female PWID. For this mapping exercise we used the broader term of transgender, as used in other similar studies although the focus was on transwomen. It should be noted that no specific information about individuals was collected, and the methodology did not physically count individuals. Rather, the methodology captured the KPs’ estimates of how many KPs are at each hotspot at various days/times.

### Data quality assurance

A team of master trainer’s including County AIDS and STI Coordinators (CASCOs), Sub County AIDS and STI Coordinators (SCASCOs), IPs and KP community leaders were trained on programmatic mapping in a three-day national level training workshop conducted by National AIDS and STI Control Programme (NASCOP), University of Manitoba (UoM), Partners for Health and Development in Africa (PHDA) and University of California, San Francisco (UCSF). At the county level, the CASCOs organized trainings of IPs including peer educators and outreach workers and led the process of field data collection. The national KP program was responsible for overall coordination, data management, analysis, monitoring of fieldwork and assuring data quality. Field supervision and data quality assurance were achieved through a multilayered monitoring mechanism. At the national level, NASCOP, UoM and UCSF monitored all field activities and conducted random supervisory visits in the field. At the county level, the CASCOs and SCASCOs along with the NASCOP site supervisors led the supervision of the mapping process.

### Data analysis

A database was designed using Open DataKit (ODK) Collect application by UCSF with both logic flows and logic checks enforced for enhanced data consistency. Data were either collected using the installed ODK collect application on Android smartphone-based or on a paper form if the interviewed subject or data collector was not comfortable with the use of an android device. Paper-based data was later captured using the ODK Collect application. Both onsite and offsite data verification was conducted by site supervisors for every 10% of data collected per day. Data managers also reviewed the data collected for consistency and completeness before data were cleaned and analyzed. If 10% or more of the data collected showed inconsistencies, the validation process was redone in those venues or hotspots. Additionally, data for MSM were revalidated based on the recommendation by the MSM community, the research and program team. They felt that the numbers of MSM need to be revalidated as some of the numbers were different from what the program generated estimates were. Thus 5%–10% of the hotspots were randomly picked within 10 randomly selected counties and were revalidated to check for under-reporting or over-reporting in those counties.

Venue or hotspot analysis and size estimates of KP at each venue or hotspot were conducted using information collected at level two. Since this information was directly obtained from KPs at the venue or hotspot level, the information had high reliability specifically regarding the numbers of KPs, their characteristics, typology and mobility. The size estimation process included finalizing the number of KPs for each venue or hotspot within an intervention site and geographic area, and aggregating it subsequently at the Ward, Sub-county, County and finally at the national level. These estimates were adjusted for the overlap as most KPs visit more than one spot, and also for the average number of spots visited by each KP. The adjustment were made independent of overlapping group participation. We agree that there are some overlapping behaviors, but we included the KP member in the group where he/she was originally counted. The following mathematical function was used i.e., E _i_= ∑ S _i_(1-P _i_)+ (S _i_*P _i_/M _i_), where S _i_= spot estimate, P _i_= proportion of KPs visiting multiple spots, M _i =_ average no of spots visited. Analysis of the data was done using SPSS version 24.0.

### Ethics and consent

The study followed all ethical principles of conducting research with human subjects including use of informed consent and maintaining confidentiality of information through strict measures. Ethical approval for this research was obtained from Kenyatta National Hospital, University of Nairobi Ethical Review Committee (ERC) of (P647/11/2017) for secondary analysis of the study data. International ethical guidance was followed to maintain confidentiality of participants i.e., no recording of participant identity or personal identification information, use of unique identifying codes and limiting access to the data files to authorized individuals only. Informed verbal consent was then obtained from the participants before the discussions started. All interviews were conducted in a safe and secure place. All participating KPs were compensated for their time and travel in Kenyan Shillings equivalent to $5 USD. Debriefing sessions were conducted after the interviews and all participating KPs were referred to HIV prevention, treatment and care facilities.

## Results

Table 1 presents the estimated KP size estimates (KPSEs) (range) of FSWs, MSMs, PWIDs and TG people in 34 counties mapped in Kenya. A total of 167,940 (129,271 to 206,609) FSWs were estimated in 10,987 geographical venues or hotspots with 9% of them being under the age of 18 years. FSWs were the largest KP in Kenya followed by an estimated number of approximately 32,580 (24,704 to 40,455) MSM, operating from 2,153 geographical venues or hotspots. Nearly 9% of these were less than 18 years old. 36% of the MSM, that is, 11,807 (8,760 to 14,854) informed that they sell sex to other men in these venues or hotspots. Approximately 16,063 (12,426 to 19,691) PWIDs were estimated at 402 venues with 11% of them being under the age of 18 years. In terms of gender, 13,658 (85%) PWIDs were male while 2,405 (15%) PWIDs were female. We also estimated 4,305 TG people from 1,218 venues. Table 1 also shows the number of KPs per 1,000 adult men or women above 18 years. We estimated 11.6 FSW per 1,000 women, 1.1 male PWIDs per 1,000 males, 0.18 female PWIDs per 1,000 females, 2.4 MSM per adult men and 0.34 transgender people per 1,000 males. This study did not include transmen, and only focused on transwomen, hence the denominator is 1000 adult men. The full dataset can be found under *Underlying data* ^24^.

**Table 1.** Size estimates of various key populations in Kenya.

Female sex workers (FSWs)	Estimated Numbers
* Estimated FSWs (range)*	167,940 (129,271 to 206,609)
* No. of venues*	10,987
* Estimated FSW below 18 years (range)*	14,809 (10,432 to 19,185)
* No. of FSWs per 1,000 adult females* **	11.6
* Average No. of FSWs per venue*	15
People who Inject Drugs (PWID)	
* Estimated PWIDs (range)*	16,063 (12,426 to 19,691)
* Estimated PWIDs below 18 years (range)*	1,831 (1,229 to 2,433)
* No. of venues*	402
* Estimated Male PWIDs*	13,658
* No. of PWID per 1000 adult men* **	1.09
* Estimated Female PWIDs*	2,405
* No. of PWID per 1000 adult women* **	0.18
* Average No. of PWID per venue*	40
Men who have sex with men (MSM)	
* Estimated MSM (range)*	32,580 (24,704 to 40,455)
* No. of venue*	2,153
* Estimated MSM below 18 years (range)*	2,949 (2,039 to 3,858)
* Estimated MSM who sell sex (Male sex workers) (range)*	11,807 (8,760 to 14,854)
* No. of MSM per 1000 adult men* **	2.37
* Average No. of MSM per venue*	15
Transgender People	
* Estimated transgender people (range)*	4,305 (2,826 to 5,783)
* No. of venue*	1,218
* No. of transgender people per 1000 adult men* **	0.34
* Average No. of transgender people per venue*	3

*Total estimated number (minimum estimate – maximum estimate) ** Number of adult females and males (18–49) in Kenya was taken from the 2019 Kenya Population Housing Census Volume III (Available at: https://www.knbs.or.ke/?wpdmpro=2019-kenya-population-and-housing-census-volume-iii-distribution-of-population-by-age-sex-and-administrative-units. Accessed April 20, 2021).

Table 2 shows information on various venues used by FSW, MSM, PWID and TG people in Kenya as well as their estimated numbers by typology of venues. A total number of 14,760 geographical venues or hotspots and locations were identified for all different KPs typologies with FSWs having the highest number of 10,987 venues or hotspots in all 34 counties. The typology of venues ranged from bars with lodging, bars without lodging, guest houses, streets, sex dens and uninhabited buildings. Bars without lodging accounted for 5,099 (46.4%) of the total FSW venues or hotspots followed by bars with lodging at 3,769 (34.3%). All the other typologies accounted for below 4% of the total FSW venues or hotspots. For MSM, a total of 2,152 venues or hotspots were mapped in 30 counties with similar typologies as FSW venues. Bars without lodging accounted for 762 (35.4%) of the total MSM venues or hotspots followed by bars with lodging (28.4%) and 192 (8.9%) street venues. All other typologies accounted for 27% of the total MSM venues or hotspots. A total of 402 venues for PWID were found in 15 counties only. These mostly included streets/highways/alleys/backstreets, injecting dens, uninhabited buildings, parks/beaches/toilets and homes. Streets/highways/alleys/backstreets accounted for 151 (37.6%) of the total PWID venues or hotspots followed by injecting dens at 130 (32.4%). All the other typologies accounted for 30% of the total PWID venues or hotspots. It was noted that people who use drugs and not necessarily injecting drugs used the same venues as those who injected drugs. Transgender people were found to use the same venues used by FSWs and MSM. A total of 1,218 such FSW and MSM venues were identified that the transgender population were also using. Nearly 3/4 of these venues included both bars with and without lodging facilities which accounted for nearly 70% of the transgender population in Kenya.

Findings indicated that KPs usually are available on the venues during the entire week and most times of the day. However, there are days specific to each KP when their numbers increase than the usual referred to as peak days and peak times. Sunday was the peak day of operation for FSWs, reported by 85% of the respondents followed by Saturday (74%). Most FSW reported being available at their specific venues on these days, especially between 6pm–10pm. Sunday and Saturday were also reported to be the peak days of operation for MSM reported by 86% of the respondents, with evenings (6pm–10pm) being the peak time of operation. Data gathered from PWIDs showed that they are present at the venues during most days of the week, with more activity seen on Fridays and Sundays mostly early mornings and evenings when PWID usually come to venues to inject drugs ( Table 2).

**Table 2.** Venue information for key populations in Kenya.

Variable	FSWs		MSM		PWID		Transgender People	
	No. of venues	Estimate	No. of venues	Estimate	No. of venues	Estimate	No. of venues	Estimate
**Venues Typology**								
Street	424	9132	192	2820	151	4913	105	397
Parks/beach/toilet	105	1945	90	1373	29	663	38	143
Residential	324	10342	114	2449	17	511	69	274
Sex den/brothel	118	2538	15	147	1	17	16	59
Strip club/ massage parlor/ salon	242	2592	29	448	2	41	15	59
Bar with lodging	3769	55839	613	8076	3	48	412	1455
Bar without lodging	5099	71012	762	12775	25	537	429	1409
Guest house	435	6912	42	430	0		36	115
Uninhabited building	44	757	12	193	39	1108	7	32
Drug dens	2	28	3	29	130	8166	2	4
Casino/club	244	4802	144	2530	5	60	63	300
Others	181	2043	137	1311	0		26	60
**Hours of operation**								
Morning (till 12 pm)	12%		5%		80%		NA	
Afternoon (12 - 5 pm)	26%		17%		27%		NA	
Evening (5 - to 9 pm)	74%		71%		41%		NA	
Night (9 pm onwards)	62%		72%		38%		NA	
**Days of** **operation**								
Monday	18%		12%		19%		NA	
Tuesday	10%		6%		28%		NA	
Wednesday	11%		8%		34%		NA	
Thursday	22%		13%		26%		NA	
Friday	53%		37%		56%		NA	
Saturday	76%		85%		36%		NA	
Sunday	85%		86%		48%		NA	

Table 3 shows an average along with minimum and maximum estimated numbers of FSWs, MSMs and PWIDs in 34 counties mapped in Kenya. Of the 167,940 FSWs estimated in all 34 counties, nearly one fourth were found in Nairobi (24%), followed by Nakuru (11%) and Mombasa (5%). More than 90% of the FSWs were found to concentrate in 24 counties. Overall, 32,580 MSM were estimated in 30 counties, of which 91% concentrated in 14 counties, with Nairobi County accounting for 31% of the total estimate. Both Kilifi and Mombasa comprised of 9% each of the total estimated MSM in Kenya. PWIDs were found in 15 counties with an estimated number of 16,063. Nearly 85% of the total PWIDs were from 4 counties, with Nairobi having the largest proportion (31%) followed by Kilifi and Mombasa, which had 27% and 16% of the estimated PWID respectively. Transgender people were the smallest number of KP with an estimated number of 4,305 transgender people found in 30 counties, they share the venues with FSW and MSM, with Nairobi County having the largest share (25%) followed by Bungoma (12%), Mombasa (10%) and Kilifi (8%) counties. 

**Table 3.** Estimated number of FSWs, MSMs, PWIDs and TGs in all Counties mapped in Kenya.

County	FSW	MSM	PWID	Transgender People
Total	167,940 (129,271–206,609)	32,580 (24,704–40,455)	16,063 (12,426–19,691)	4,305 (2,826–5,783)
Bomet	3,309 (2,585–4,032)	120 (100–140)	NA	9 (4–14)
Bungoma	3900 (2,699–5,100)	1,562 (1,162–1,961)	NA	518 (336–699)
Busia	2,968 (2,408–3,527)	572 (440–704)	NA	NA
Embu	1,851 (1,332–2,369)	132 (93–171)	NA	NA
Homa bay	3,783 (2,594–4,971)	252 (176–327)	91 (55–127)	87 (56–118)
Kajiado	7,642 (6,359–8,924)	474 (311–636)	63 (43–83)	113 (71–155)
Kakamega	1,751 (1,352–2,150)	637 (487–787)	NA	91 (65–117)
Kericho	2,333 (1,937–2,728)	NA	NA	10 (8–12)
Kiambu	5,810 (4,780–6,839)	1,664 (1,264–2,064)	1,230 (1,045–1,415)	93 (61–124)
Kilifi	6,696 (4,963–8,428)	2,868 (2,064–3,671)	4,308 (3,168–5,447)	341 (218–463)
Kirinyaga	2,497 (1,858–3,135)	15 (10–19)	NA	2 (1–3)
Kisii	6,538 (4,908–8,168)	462 (328–595)	36 (29–43)	62 (39–85)
Kisumu	5,151 (3,894–6,407)	2,492 (1,764–3,220)	491 (390–592)	228 (140–316)
Kitui	2,856 (2,164–3,547)	44 (34–54)	NA	37 (21–53)
Kwale	2,833 (2,051–3,615)	681 (540–821)	1,736 (1,127–2,336)	68 (45–91)
Laikipia	1,154 (818–1,489)	138 (91–185)	NA	185 (128–241)
Machakos	4,916 (4,050–5,782)	2,234 (1,546–2,921)	57 (47–67)	275 (193–357)
Makueni	2,743 (2,218–3,268)	338 (227–448)	NA	NA
Meru	2,515 (2,098–2,932)	55 (37–72)	75 (60–89)	8 (5–11)
Migori	4,709 (3,548–5,869)	559 (372–745)	202 (153–250)	183 (86–279)
Mombasa	8,187 (6,016–10,357)	2,855 (2,291–3,418)	2,591 (1,992–3,189)	435 (300–569)
Muranga	2,533 (2,142–2,142)	NA	NA	2 (2–2)
Nairobi	39,643 (31,146–48,139)	10,209 (8,200–12,217)	5,024 (4,198–5,849)	1,064 (688–1,439)
Nakuru	17,708 (13,278–22,138)	2,072 (1,438–2,706)	23 (9–36)	82 (59–105)
Narok	3,064 (2,383–3,745)	59 (44–73)	NA	11 (9–12)
Nyamira	1,999 (1,432–2,566)	107 (80–133)	NA	3 (2–3)
Nyeri	1,299 (1,060–1,537)	123 (108–137)	NA	10 (7–12)
Siaya	4,027 (3,087–4,967)	663 (485–841)	110 (87–132)	95 (69–120)
Taita taveta	1,611 (1,163–2,059)	NA	NA	15 (9–20)
Tharaka nithi	2,594 (1,972–3,215)	141 (107–175)	NA	15 (7–23)
Trans nzoia	2,522 (1,724–3,320)	NA	NA	1 (1)
Turkana	3,722 (2,890–4,553)	450 (383–517)	NA	183 (138–228)
Uasin gishu	2,886 (2,202–3,570)	83 (68–97)	30 (23–36)	46 (34–57)
Vihiga	200 (160–240)	527 (454–600)	NA	40 (25–54)

**Figure 1.**
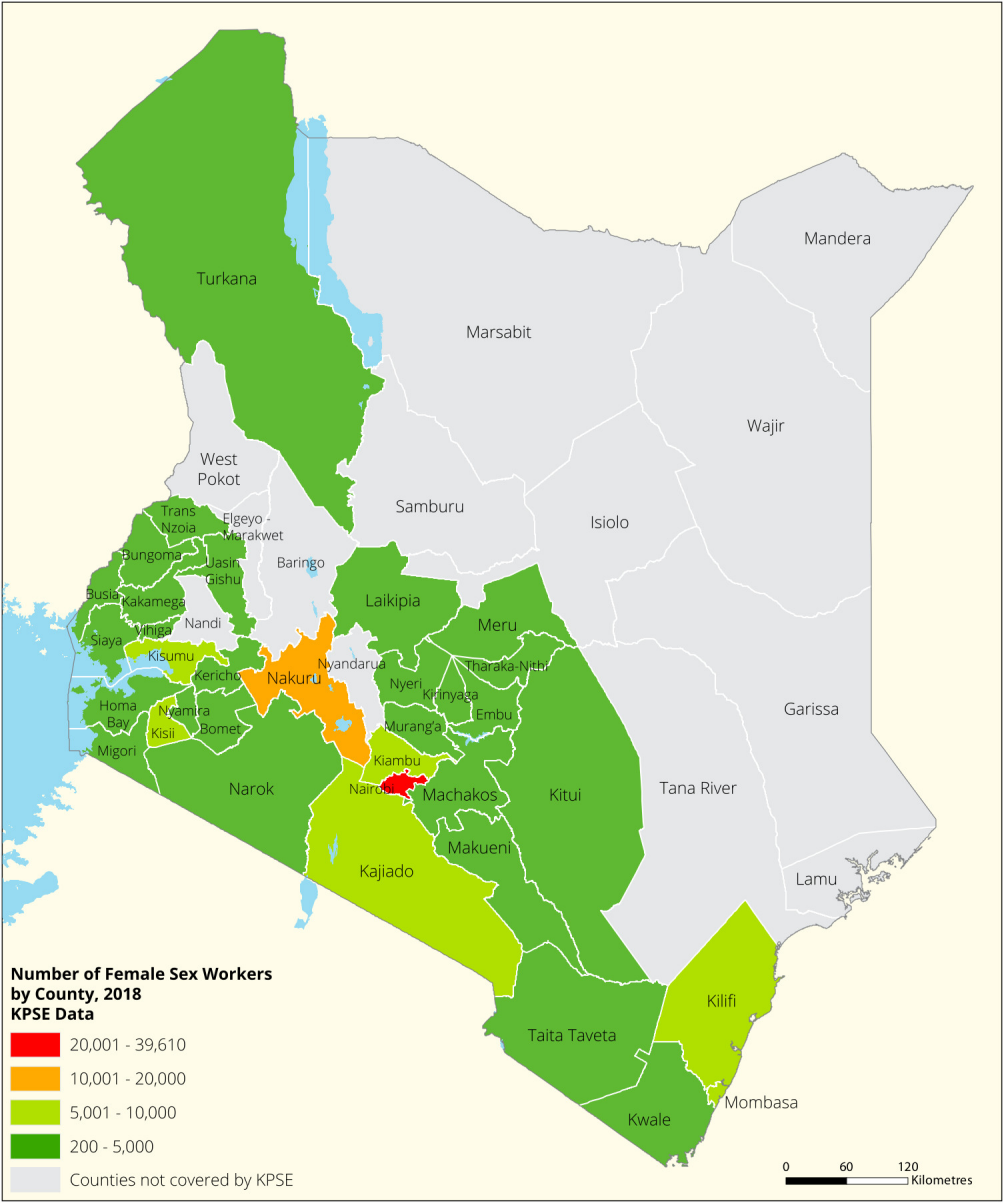
Distribution of FSWs mapped in different counties in Kenya.

**Figure 2.**
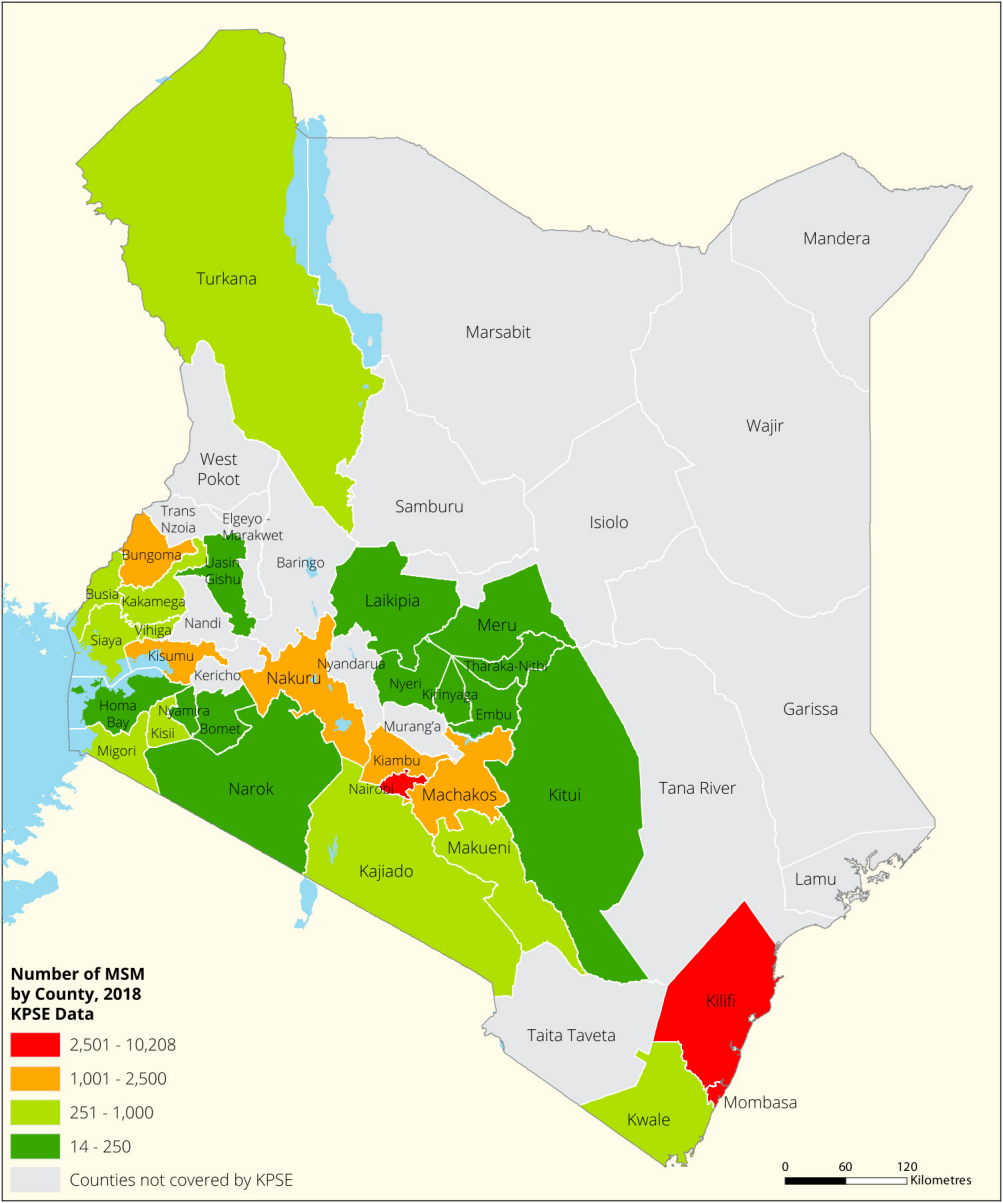
Distribution of MSMs mapped in different counties in Kenya.

**Figure 3.**
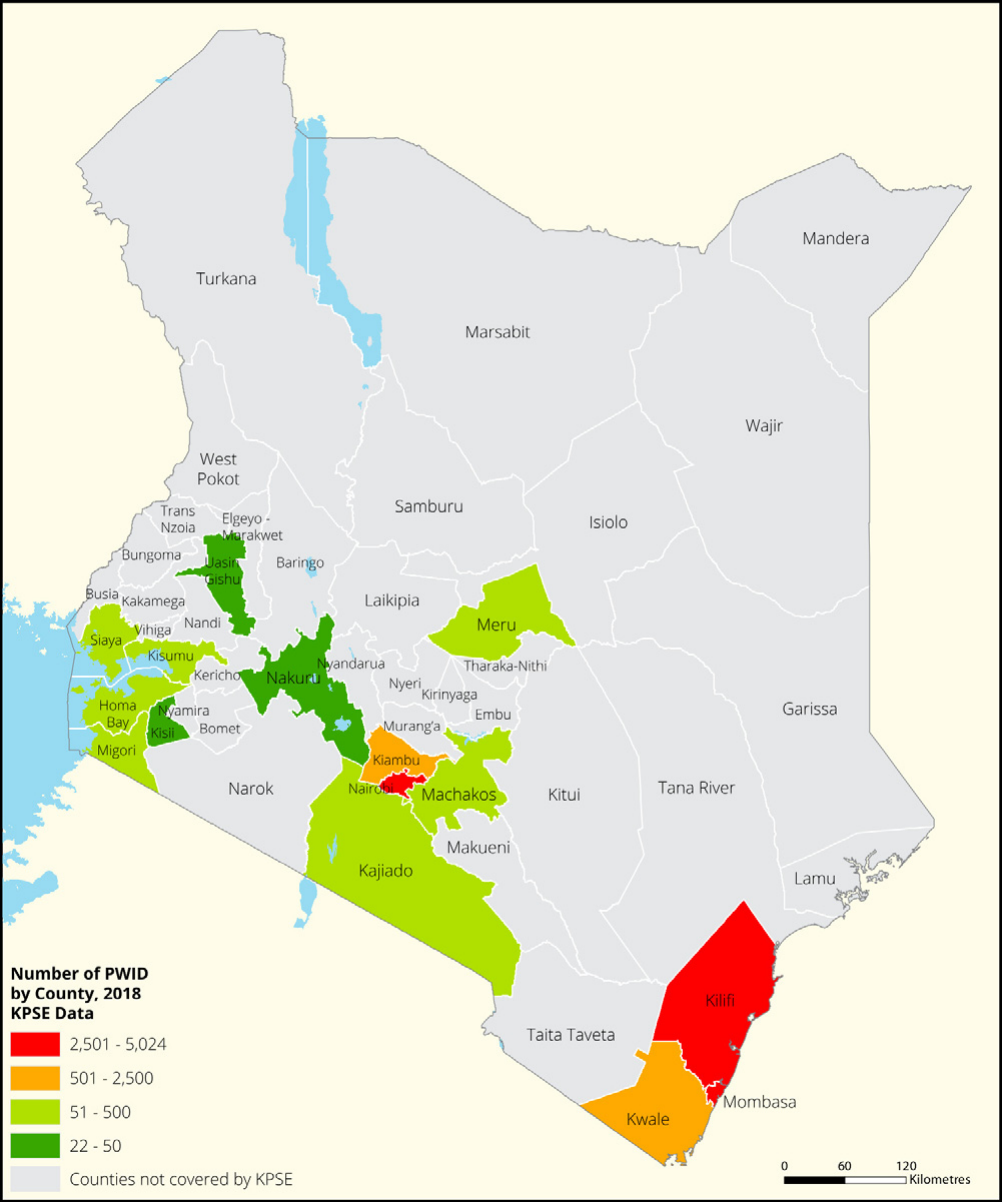
Distribution of PWIDs mapped in different counties in Kenya.

**Figure 4.**
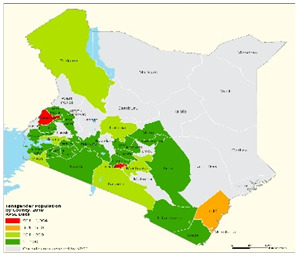
Distribution of TG people mapped in different counties in Kenya.

Figure 1, Figure 2, Figure 3 and Figure 4 shows the distribution of FSWs, MSMs PWIDs and Transgender People in the different counties in Kenya. As shown FSWs were present in all 34 counties, MSM in 30 counties PWIDs in 15 counties and TG people were only found in 30 out of the 34 covered by the exercise.

## Discussion

Programmatic mapping and KP size estimates were first conducted in Kenya in 2012 ^25^. Data generated from mapping have been used effectively by NASCOP to develop HIV prevention strategies and service delivery for KPs since 2012 ^21^. This study not only contributed to updating the KP national size estimates, it generated evidence of young KPs and also provided county wise estimates in 34 out of the 47 counties in Kenya to revise program indicators. Furthermore, this was the first time that Kenya has estimated the number of transgender people and provided evidence for their inclusion as a KP in the Kenya AIDS Strategic Framework II 2020/21-2024/25 ^18^.

Comparing the size estimates of KPs from 2012 ^25^, we have noticed a 26% increase in the overall estimated number of FSW (133,675 to 167,940) and a 76% increase in MSM (18,460 to 32,580), while the number of PWID has decreased from 18,327 to 16,063. This reduction in the number of PWIDs could be due to the scale-up of the medically assisted therapy (MAT) program in the country in the last three years ^26^. The higher number of FSWs and MSM reported could be due to an improved mapping methodology and size estimation technique as well as using the KPs to report on additional hotspots that they knew and were eventually also mapped. The number of KPs estimated through this exercise were calculated using information obtained from KPs frequenting venues and might be an underestimate of the total population of KPs in the country. This is because we would have missed those KPs who only frequent virtual sites or do not visit venues to find sex partners. A virtual mapping conducted in three counties for MSM showed that 75% of the MSM who mostly use virtual sites to find partners also visit physical venues ^23^. Thus the overall estimated number of MSM in Kenya might be 20% to 25% higher than the numbers presented in this study. It is also important to note that a high proportion of MSM work as male sex workers and should be the focus of HIV prevention activities in Kenya.

It is worth mentioning that while literature consistently documents limited success in estimating and reaching young KPs ^27^, this study was able to document a substantially higher proportion of young KPs among all groups mapped. An estimated 14,809 FSWs (9%), 2,949 MSM (9%) and an estimated 1,831 PWID (11%) were younger than 18 years of age which shows that Kenya needs to strategize tailored care and service delivery programs to increase young KP engagement in HIV services. We also found a significant mixing of key populations in multiple venues. For example, we saw transgender people using the same venues used by FSWs and MSM. In addition, we saw that PWID venues were also used by people who use drugs without injecting. We also found a significant number of females who inject drugs in venues used by males who inject drugs. The PWID program had challenges in getting female PWIDs to come in for services and based on the data, they do share the same hotspots, it is imperative then to design our programs to ensure our services appeal to the female PWID such as having female PWID only days/times for services and inclusion of care packages.

From a program perspective, this study has also provided valuable information to the HIV prevention program for key populations in Kenya, about counties which need to be prioritized to reach KPs and appropriate use of limited resources for the most effective coverage. Eighteen counties out of all the counties mapped in Kenya, collectively contained 80% of all FSWs mapped. That is 79,876 out of 167,940 FSWs. For MSM, eight out of all counties mapped collectively accounted for 80% (25,956 of the 32,580) of the total estimated number of MSM. Likewise, for PWID, 15 counties collectively accounted for 85% of the estimated PWID and, for transgender people, 10 counties out of all the counties mapped accounted for 82% of the estimated population. With limited resources, these data can help the country prioritize KPs in these counties for optimal coverage and effective utilization of resources.

The approach presented in this paper has several strengths. One of the key strengths is the leadership role played by the national and county governments and ownership of data collected. The rapidity and simplicity of the process and strong involvement of the KP in the development and implementation of the approach also need mentioning. Data were collected by KP members including peer educators and outreach workers who knew about the venues where KP congregate, and later used this information to provide services.

The exercise also had some limitations. The estimates generated are not based on any physical counting of individuals, rather they are dependent on the reporting of the respondents. Thus, there could be an under-estimation of some KP sub-populations at the venue level. Moreover, KPs that find partners online and do not usually come to these physical locations were likely to be missed and not counted. KPs that frequented multiple venues were likely to be enumerated more than once. Although in the final analysis, this duplication was adjusted for, there still is a possibility of double counting some individuals especially if they use different typology of venues. To overcome these calculation issues, we propose that the country must triangulate data from multiple sources e.g., program data and finalize the size estimates of these KPs mapped. Another limitation was that we did not interview transgender people but collected their data through FSW and MSM. This means that data collected maybe not applicable for transgender people, as it was not collected from them. Finally, We are also cogniscant of the fact that social gatherings for KPs are not just to find sexual partners, but as a way of networking and soacializing. This programmatic mapping however, was conducted to identify populations that are actively seeking partners at venues (FSW/MSM and TG) and/or congregating for injecting (PWIDs).

Despite limitations, this study was able to provide robust information on the size, locations and operational dynamics of KPs with a good indication of where HIV services could be provided for effective control of HIV. These revised estimates will be able to provide new targets and resources for the KP program in Kenya and will also help the government, donors and implementers monitor the effective coverage of existing key population programs in various geographies and populations.

Finally, to conclude, this study not only provides updated estimates of various KP mapped but also brings context-specific epidemiological evidence which is highly beneficial to guide HIV prevention program development and implementation ^26^. The country must set up systems and interventions to reach out to those more difficult to reach and use this information to develop high impact interventions for effective control of HIV in Kenya.

## Data Availability

Data on the Number of adult females and males (18–49) in Kenya was taken from 2019 Kenya Population Housing Census Volume III (Available at: https://www.knbs.or.ke/?wpdmpro=2019-kenya-population-and-housing-census-volume-iii-distribution-of-population-by-age-sex-and-administrative-units. Accessed April 20, 2021). This data is confidential considering the fact that KPs are a criminalized population in Kenya and sharing names of sites may put their life in danger. The raw data from the interviews is in SPSS format. Aggregate level de-identified data tables are available on Harvard Dataverse (see below). The corresponding author ( jmusimbi@gmail.com) will be able to facilitate access to the full underlying data. A formal request needs to be made and a data sharing agreement will have to be made before sharing the data. Harvard Dataverse: Data of Programmatic mapping and estimating the size of Female sex workers, Men who have sex with men and People who inject drugs, in Kenya: https://doi.org/10.7910/DVN/ZO5T7Z ^24^. This project contains the following underlying data: -  Data Dictionary.xlsx -  KPSEdata.tab Harvard Dataverse: Data of Programmatic mapping and estimating the size of Female sex workers, Men who have sex with men and People who inject drugs, in Kenya: https://doi.org/10.7910/DVN/ZO5T7Z This project contains the following extended data: Form Validation form.docx (Form B) Data are available under the terms of the Creative Commons Zero "No rights reserved" data waiver (CC0 1.0 Public domain dedication).
