# Supplementary material for: Programmatic mapping and estimating the population size of female sex workers, men who have sex with men, people who inject drugs and transgender populations in Kenya
Source: Gates Open Res. 2024 Aug 22;6:112. Originally published 2022 Aug 22. [Version 4] doi: 10.12688/gatesopenres.13623.4 (PMC11259589; doi:10.12688/gatesopenres.13623.4)
Supplement: Peer review history

# Reviewer report: Reviewer response for version 4

**DOI:** 10.21956/gatesopenres.17681.r39208

**Published:** 2025-01-23

**Authors:** Daniel McCartney

**Affiliations:** 1 London School of Hygiene & Tropical Medicine, London, UK

This article provides an in-depth summary of a programmatic mapping and size estimation exercise for key populations (KPs) in Kenya conducted in 2018. The research focuses on female sex workers (FSWs), men who have sex with men (MSM), people who inject drugs (PWIDs), and transgender women. The article, though based on data from several years ago, is relevant for Kenya’s future efforts in KP size estimation and are useful for other countries planning similar exercises.

 Importantly, this study is notable for its inclusion of transgender women in the mapping and estimating process and its age differentiations for KPs (including those under 18 years of age) and gender differentiation for PWIDs and sex workers. This differentiation enhances the specificity and usability of the data for policymaking and programmatic interventions. This version is mostly complete but would benefit from further editing to ensure consistency and improved readability, and considering the following overall and section-specific comments:

**Overall comments:**

 This version demonstrates efforts to specifically and accurately detail the inclusion of transgender women in the size estimation and mapping. To ensure consistency and clarity, I recommend revising the title to explicitly mention transgender women and ensuring uniform use in text and in tables and text (e.g., replace "transgender people" with "transgender women" in Table 1, as done for Table 2 & 3). Although correct in the few instances used, I would also suggesting replacing "transwomen" with "transgender women" for consistency and clarity.

**Section-specific comments:**

*Introduction:*

 -Suggest to indicate the year of HIV prevalence estimates for the listed KPs (although note reference is from 2015), and the year when the additional size estimate was completed in Nairobi.

 -For clarity, suggest to review statement that this study represents the first time size estimates for transgender women were conducted in Kenya. Suggest to define transgender women explicitly (e.g., "those assigned male sex at birth who identify as female") to avoid ambiguity.

 -Suggest to state that this is the first KP size estimation to provide age differentiation for all populations, and gender distinctions for PWIDs and sex workers in Kenya.

 -Suggest to conclude the introduction with a clearer statement of the study’s aims.

*Methods:*

 -Suggest to clarify whether the size estimation of transgender women was a distinct process or integrated into the MSM and FSW exercises. Similarly, ensure to clearly specify that male sex workers (MSWs) were estimated solely through the MSM exercise. This is mentioned in the limitations, but would be useful in the methodology.

 -As noted by other reviewer, clarify whether the 25% of MSM not visiting physical locations were included or excluded in the estimates.

*Results:*

 -Suggest to review and correct numerical expressions (e.g., "2.4 MSM per adult men" should read "2.4 MSM per 1,000 adult men"). Ensure that all values are presented comprehensively with minimum and maximum ranges.

 -Suggest to revise terminology in the results section for greater accuracy and to minimize potential stigmatization. For example, when describing when present at specific venues, suggest to replace "peak days of operation" for MSM with "peak days of activity" or similar (as done for PWIDs). Also, as providing size estimate specifically for transgender women, possibly state as "per 1,000 adults assigned male sex at birth" rather than "per 1,000 adult men".

 -Ensure to maintain similar clarity in Figures 1-4 visualizing the distribution of KPs (Fig 4 is not clear).

*Discussion:*

 -Suggest to expand on the positive outcomes of the study, such as the inclusion of transgender people in the updated Kenya AIDS Strategic Framework (KASF). Are there additional applications or benefits resulting from this work?

 -Suggest to discuss whether Kenya has plans to update the KP size estimations, given the previous KASF recommendation to revise these every five years.

**Minor comment:**

 I suggest to refer to the 2024 UNAIDS terminology guidelines (https://www.unaids.org/en/resources/documents/2024/terminology_guidelines) to ensure inclusive and appropriate terminology to avoid use of potentially stigmatizing language. For example, "use of unsterile injecting equipment" rather than "sharing contaminated needles".

Is the work clearly and accurately presented and does it cite the current literature?

Partly

If applicable, is the statistical analysis and its interpretation appropriate?

I cannot comment. A qualified statistician is required.

Are all the source data underlying the results available to ensure full reproducibility?

Yes

Is the study design appropriate and is the work technically sound?

Yes

Are the conclusions drawn adequately supported by the results?

Partly

Are sufficient details of methods and analysis provided to allow replication by others?

Yes

Reviewer Expertise:

Clinical epidemiology of HIV and other STIs among key populations

I confirm that I have read this submission and believe that I have an appropriate level of expertise to confirm that it is of an acceptable scientific standard, however I have significant reservations, as outlined above.

## References

[1] UNAIDS Terminology Guidelines. *Geneva: Joint United Nations Programme on HIV/AIDS* .2024; Reference source

# Reviewer report: Reviewer response for version 4

**DOI:** 10.21956/gatesopenres.17681.r39129

**Published:** 2025-01-07

**Authors:** Rebecca Anderson

**Affiliations:** 1 Imperial College London, London, England, UK

This article summarises the outputs from a key population programmatic mapping exercise conducted in Kenya in 2018. The study extends and updates the mapping exercise conducted in the country in 2012 offering a more comprehensive overview of KP programming needs in the country. This article is largely complete, and a useful addition to the literature, but would benefit from elaboration and editing in some places.

 A minor comment is to focus on ensuring consistency when using of abbreviations.

 It would also be useful to define the size estimate bounds (e.g. as min and max estimates) at first reporting in the abstract and results for ease of interpretation. 

 Section-specific comments as follows: 

Introduction:

 The background literature on countries with KP size estimates could be updated. A recent review by Stevens et al identified estimates for a larger number of countries than the existing citation (Supplement Table 3 from Stevens 2024).

 The final sentence of the introduction, identifies “comprehending changes in KP dynamics over time” as an aim  - this feels vague and would benefit from clarifying exactly what is being investigated here. 

Methods:

 In the description of the Level One stage, it may be useful to describe how KP informants were selected to contribute to the consulting process to understand where bias was avoided, or may have been introduced. 

 The methods discuss scaling up the MSM PSEs by 25% to account for individuals seeking partners virtually. However, in the discussion, there is the statement “Thus the overall estimated number of MSM in Kenya might be 20% to 25% higher than the numbers presented in this study”, which seems to conflict the methods. Please clarify whether or not an adjustment was applied. 

Results:

 The number of venues for each KP do not match the numbers listed in the abstract. 

 The TG size estimate is also missing min/max bounds. 

Discussion:

 Regarding the comments surrounding difficulty engaging female PWID in programs, it may be useful to provide an estimate of the ratio between male and female PWID engaged in programmes to compare against the number mapped, as there are still considerably fewer female injectors estimated, relative to men. 

 Updating the 2012 mapping exercise is undoubtedly useful, but it may also be useful to discuss these results in the context of the Phase II PSE exercise finalised in 2020 - how do these mapping estimates compare to the other methods implemented?

Is the work clearly and accurately presented and does it cite the current literature?

Partly

If applicable, is the statistical analysis and its interpretation appropriate?

I cannot comment. A qualified statistician is required.

Are all the source data underlying the results available to ensure full reproducibility?

Yes

Is the study design appropriate and is the work technically sound?

Yes

Are the conclusions drawn adequately supported by the results?

Partly

Are sufficient details of methods and analysis provided to allow replication by others?

Partly

Reviewer Expertise:

HIV epidemiology in key populations

I confirm that I have read this submission and believe that I have an appropriate level of expertise to confirm that it is of an acceptable scientific standard, however I have significant reservations, as outlined above.

## References

[1] : Population size, HIV prevalence, and antiretroviral therapy coverage among key populations in sub-Saharan Africa: collation and synthesis of survey data, 2010-23. *Lancet Glob Health* .2024;12(9) : 10.1016/S2214-109X(24)00236-5 e1400-e1412 10.1016/S2214-109X(24)00236-5 39151976 PMC11345451

# Reviewer report: Reviewer response for version 3

**DOI:** 10.21956/gatesopenres.16399.r36634

**Published:** 2024-07-19

**Authors:** Rajatashuvra Adhikary

**Affiliations:** 1 World Health Organization -India Country Office, New Delhi, Delhi, India

Overall, I found it as an excellent piece of work. The methodology is pretty comprehensive and pragmatic. Both language as well as flow of the paper is simple but very convincing. I am considerably impressed with this work. It will make significant contributions to the existing knowledge base in this technical area or domain. However, I do have few observations, inputs,  queries and suggestions to share for authors' consideration:

 1. An appropriate abbreviation for 'programmatic mapping and size estimation' could be 'p-MPSE'. 

 2. The sub-title 'Sampling' may not be appropriate in the context of this exercise. In fact, it is the 'Coverage' of the p-MPSE in Kenya during 2018.

 3. In 'Abstract', please refer to a specific figure mentioned in the second line of 'Results' as '10,951 (8,160 6o 13,742)' - It is not the estimated number of FSW or MSM or PWID or TG.  Then, what is it?

 4. Generally at the hotspot level, In-depth Interviews with Key Informants are preferred to collect the necessary information due to several advantages. Why FGDs were conducted in this exercise is not clear and could be explained properly. 

 5. It is mentioned in the text that the participants for the FGD were 'randomly' selected at the hotspot level. Why and how we did so need to be explained. In fact, we should be a bit careful in using the word 'randomly'.

 6. Generally, in other p-MPSE, we estimate maximum and minimum numbers at the hotspot level. It is not clear why this exercise had three estimates (low, mid and high) for each hotspot. How was it beneficial?

 7. In page 6, it is mentioned that there was 2.4 MSM per adult men. It must be a very serious typo. Let's correct it immediately. 

 8. Table 1 : It will be good to reflect the percentage of female PWID (which is 15%) beside absolute number.

 9. Table 2: Why 'hours of operation' and 'days of operation' are missing in case of TG population?

 10. It seems most of the TG venues are 'mixed venues' (mixed with FSW or MSM venues). However, may be, Nairobi may have some exclusive TG venues (bars where only TGs are soliciting). Do we have that?

 Anyway, these are very minor observations and comments. Once again, I highly appreciate this excellent piece of work. It must be indexed soon.

Is the work clearly and accurately presented and does it cite the current literature?

Yes

If applicable, is the statistical analysis and its interpretation appropriate?

Yes

Are all the source data underlying the results available to ensure full reproducibility?

Yes

Is the study design appropriate and is the work technically sound?

Yes

Are the conclusions drawn adequately supported by the results?

Yes

Are sufficient details of methods and analysis provided to allow replication by others?

Yes

Reviewer Expertise:

Research, Surveillance and Strategic Information including routine program monitoring, evaluation etc.

I confirm that I have read this submission and believe that I have an appropriate level of expertise to confirm that it is of an acceptable scientific standard.

# Response

**Published:** 2024-08-21

**Authors:** Janet Musimbi

**Affiliations:** Partners for Health and Development in Africa, Kenya

Dear Mr. Rajatashuvra Adhikary,

 Thanks a lot for reviewing the manuscript “Programmatic mapping and estimating the population size of female sex workers, men who have sex with men, people who inject drugs and transgender populations in Kenya.”

 We would like to acknowledge and thank you and editors for their positive reviews and comments. We have had discussion with our authoring team and have revised the manuscript based on the changes proposed. Please find enclosed our response to the reviewer’s comments.

 Thanks a lot again and we remain with our kindest regards

 JANET.

**REVIEWER 01**

**COMMENTS**

**RESPONSE AND REVISIONS MADE**

 1

 An appropriate abbreviation for 'programmatic mapping and size estimation' could be 'p-MPSE'. 

 Thank you for this suggestion. We however request to maintain the use of “Programmatic mapping and Size Estimation” as this is a term that is commonly in use and easily identifiable with programs.

 2

 The sub-title 'Sampling' may not be appropriate in the context of this exercise. In fact, it is the 'Coverage' of the p-MPSE in Kenya during 2018.

 Similarly to the response above we request to maintain the use of “Programmatic mapping and Size Estimation”

 3

 In 'Abstract', please refer to a specific figure mentioned in the second line of 'Results' as '10,951 (8,160 to 13,742)' - It is not the estimated number of FSW or MSM or PWID or TG.  Then, what is it?

 Thank you for this and this has been corrected.

 This was the estimated number of People who use drugs but since the focus was on PWIDs, we will focus on them and delete this.

 4.

 Generally at the hotspot level, In-depth Interviews with Key Informants are preferred to collect the necessary information due to several advantages. Why FGDs were conducted in this exercise is not clear and could be explained properly. 

 We focused on the peers to provide hotspot wide information in a manner that engages the members of that hotspot. So, FGDs helped peers to share their knowledge and insights about the hotspot and eventually agreed on the different aspects of the questions including the number of people who congregate there.

 5.

 It is mentioned in the text that the participants for the FGD were 'randomly' selected at the hotspot level. Why and how we did so need to be explained. In fact, we should be a bit careful in using the word 'randomly'.

 The approach was that once the mapping team approached a particular hotspot, with the help of the peer educator for that hotspot, approached peers who were in the hotspot at that time and requested them if they wished to participate in the FGD. This ensured that the team was heterogenous in terms of age and risk behavior  and therefore becoming  representative.

 6.

 Generally, in other p-MPSE, we estimate maximum and minimum numbers at the hotspot level. It is not clear why this exercise had three estimates (low, mid and high) for each hotspot. How was it beneficial?

 In this exercise we had low and high numbers of KPs at the hotspot. The mid is the average number of the KPs and this was used to as the estimated number of KPs that were then populated for the country.  This is beneficial in planning and target setting for programs. 

 7.

 In page 6, it is mentioned that there was 2.4 MSM per adult men. It must be a very serious typo. Let's correct it immediately. 

 This figure is per 1000 men and not a percentage. It is based on the population of adult male as per 2019 Kenya Population Housing Census Volume III.

 8.

 Table 1: It will be good to reflect the percentage of female PWID (which is 15%) beside absolute number.

 Thank you for this and it has been included.

 9.

 Table 2: Why 'hours of operation' and 'days of operation' are missing in case of TG population?

 Thanks for highlighting the missing information for Transgender populations. We cannot say that the peak day and time concept is not applicable for transgender people, as it was not collected from them. This could be one of the limitations and we have now mentioned this in the limitations.

 10.

 It seems most of the TG venues are 'mixed venues' (mixed with FSW or MSM venues). However, may be, Nairobi may have some exclusive TG venues (bars where only TGs are soliciting). Do we have that?

 For this specific study, exclusive TG venues were not identified. However, there was a follow mapping exercise that was carried out for the country and exclusive TG venues were also identified.

# Reviewer report: Reviewer response for version 2

**DOI:** 10.21956/gatesopenres.16246.r34754

**Published:** 2023-09-05

**Authors:** Denton Callander, Jonathan Odingo

**Affiliations:** 1 The Kirby Institute, University of New South Wales, Sydney, New South Wales, Australia; 2 Médecins Sans Frontières, Mombasa, Kenya

To the authors' reply, we make the following comments. These relate to how populations were conceived in this study and from our perspective they represent significant conceptual flaws in the current description of this study, which can be rectified but only with further semantic clarification in the manuscript.

**Author Report:**

 This study did not include transmen, and only focused on transwomen, hence the denominator is 1000 adult men.  For this mapping exercise we used the broader term of transgender, as been used in other similar studies. As per your suggestion, we have now indicated this in the manuscript, but would like to continue using the term Transgender.

**Reply:**

 It is fine to use the term ‘transgender’ but without specifying this study’s interest in ‘transgender women’ then it becomes meaningless and inappropriate (especially in the context of adult men as a denominator).

 In the strongest terms, we encourage the authors to specify throughout that this is a study of transgender women rather than transgender people broadly. The exclusion of transgender men should also be explained as a limitation of the method.

**Author Report:**

 As already mentioned, this study focused on transmen and Lesbian/Transgender women were not included. When at the hotspot we only focused on the population defined. We had Social mobilizers from these hotspots involved in the exercise and we had definitions of these populations. Different levels of data collection were done and the social mobilizers had a clear instruction on which type of KPs to interview.

**Reply:**

 The point in our original comment has been somewhat missed; it is about the likely overlap of key populations. How does the method deal with a sex worker who uses drugs? Why is the likely overlap never mentioned? The conceptualisation of so-called ‘typologies’ seems to assume that one can only be classified as one ‘key pop’ at a time; how was the dealt with in the method?

Is the work clearly and accurately presented and does it cite the current literature?

Partly

If applicable, is the statistical analysis and its interpretation appropriate?

Yes

Are all the source data underlying the results available to ensure full reproducibility?

Partly

Is the study design appropriate and is the work technically sound?

Yes

Are the conclusions drawn adequately supported by the results?

Partly

Are sufficient details of methods and analysis provided to allow replication by others?

Partly

Reviewer Expertise:

NA

We confirm that we have read this submission and believe that we have an appropriate level of expertise to confirm that it is of an acceptable scientific standard, however we have significant reservations, as outlined above.

# Response

**Published:** 2023-10-13

**Authors:** Janet Musimbi

**Affiliations:** Partners for Health and Development in Africa, Kenya

Dear Mr. Denton and Mr. Jonathan,

 Thanks a lot for reviewing the manuscript “Programmatic mapping and estimating the population size of female sex workers, men who have sex with men, people who inject drugs and transgender populations in Kenya.”

 We would like to acknowledge and thank you and editors for their positive reviews and comments. We have had discussion with our authoring team and have revised the manuscript based on the changes proposed. Please find enclosed our response to the reviewer’s comments.

 Thanks a lot again and we remain with our kindest regards

 JANET.

**RESPONSES**

 1

 It is fine to use the term ‘transgender’ but without specifying this study’s interest in ‘transgender women’ then it becomes meaningless and inappropriate (especially in the context of adult men as a denominator).

 In the strongest terms, we encourage the authors to specify throughout that this is a study of transgender women rather than transgender people broadly. The exclusion of transgender men should also be explained as a limitation of the method.

*Thank you for this suggestion and the paper has been revised to include that the study’s focus was transgender women and the exclusion of transgender men included in the limitations.*

 2

 The point in our original comment has been somewhat missed; it is about the likely overlap of key populations. How does the method deal with a sex worker who uses drugs? Why is the likely overlap never mentioned? The conceptualisation of so-called ‘typologies’ seems to assume that one can only be classified as one ‘key pop’ at a time; how was the dealt with in the method?

*Thank you for this clarification. The study was not able to identify the overlap of typologies among the key population and this has been documented as a limitation of the study.*

# Reviewer report: Reviewer response for version 1

**DOI:** 10.21956/gatesopenres.14899.r33412

**Published:** 2023-07-05

**Authors:** Minja Milovanovic

**Affiliations:** 1 University of the Witwatersrand Johannesburg, Johannesburg, Gauteng, South Africa

General Comments

 The current article outlines the approach and outcomes of the 2018 programmatic mapping and size estimation for key populations in 34 counties in Kenya. The mapping processes allowed for the validation of existing site and size estimations. The populations that were mapped included: female sex workers, MSM, PWID and transgender people.

 The authors, their partners and collaborators need to be acknowledge for the very important work done. Programmatic mapping and size estimations are crucial for understanding the scope of key populations and prioritizing their health needs.

 Overall the manuscript is well written, concepts are clearly defined and the rationale well established.

 The below points serve to help the authors refine their manuscript and point where additional information is required.

Abstract

- In the concluding part of the abstract there is mention that the study provided information on young KP the abstract also mentions Female PWID – did the authors intend to highlight ‘young’ key pop and specifically Female PWID despite this not being the main focus of the manuscript?

Introduction 

- In the first part of the introduction the authors outline 3 points for why programmatic mapping and size estimations are important: “helps to estimate population size…” What are the other benefits of mapping and size estimations for the programmes apart from retention?
- As this is the first time that a size estimation on the transgender population is being done, some additional information in the introduction would be beneficial.
- What type of interventions (public health, clinical?) were scaled up in the 34/47 counties post the 2012 mapping and size estimation.
- Provide some additional information on why mapping and size estimations were only done in the 34 counties where KP programmes were being implemented. Is it cause there are no programmes in the other counties and if so some insight into why that is the case. Does it have anything to do with the previous mapping following which interventions were implemented in the 34 counties. Is the idea to update size and understand changes in the initial 34 sites only or potentially also expand. 
- Last section of the Introduction: mapping and size estimation are highlighted as distinctly different steps however there seems to be a sentence missing that links the two steps.

Methods

 Overall the methodology is well implemented and involved relevant stakeholders, who were trained and outputs were monitored. However, there were some points that required additional clarity

- Were all hotspots (past and new) physically validated and if so how did the teams (implementing partners) achieve that in a 3 month period especially when there were over 10 000 female SW venues? Was the mapping, validation and size estimates completed in 3 months?
- Define operational typologies – does this refer to venue type such as bar etc?
- Need clarity regarding programme selection – of the 34 counties only those that provided services to specific KPs were selected. For example, of the 34 counties, 15 provided services for PWID and hence only 15 were included? 
- The authors mention that estimates were adjusted to account for the overlap of KPs visiting more than one spot. How did authors account for duplicate counting/estimations such that female SWs provide one estimate and PWID provide another estimate but the groups might overlap? 
- In level 2: how were the 3-5 KP members that partook in FGDs identified?
- Were the 3-5 members all part of the same KP group and were the KPs interviewed at the same time or separately?
- Who identified the gatekeeper and how were gatekeepers approached to ensure buy in?
- Was any method used to guide KP member estimates
- For data that was being captured via paper form, what was the time frame for electronic data capture?
- Why was the MSM data revalidated in 10 counties?
- Was only verbal consent obtained and if so explain why this was sufficient if participants were debriefed and referred following participation? Also, why were participants debriefed and around what? Is the referral to care facilities standard of care for all KPs or was it specific to only those that took part in the study?
- Did FGDs participants provide a percentage (eg. 9%) of KPs who were younger than 18 years or did they give an estimated value?

Results and Discussion

- Table 2 is divided into two parts with one part providing values while the second part provides percentages. How were the percentages calculated for hours and days of operation. Explain why hours and days of operation were not applicable for transgender people.
- How many of the KP venues overlapped? When aggregated by KP type there were a total of 14760, it was noted in the paper that key pops such as transgender people shared venues/hotspots with female SW and MSM.
- Under methods it is described that 30 counties were selected for mapping transgender people however the results report on information from 32 counties. Explain the inclusion of the additional counties.
- Include percentages in table 3 for ease of reading.
- Why is there no map of the transgender population?
- In the discussion the authors highlight that a high number of MSM also work as male SW. Is there any data from the mapping process to show this.
- Explain the significance of finding females and males who inject drugs sharing venues? 
- In the discussion, bring in the other counties for which there are no mapping and size estimations – is there any potential to gather data from these places
- The authors cannot say that 18 counties in the whole of Kenya, rather it’s 18 counties of all those that have been mapped.
- If the authors mapped 15 counties for PWID but only account for 85% of the cohort please explain the difference?
- How did the KP members (peer educators and outreach workers) use the information to provide services and what services?
- What was the total number of respondents that the estimates are based on?

Is the work clearly and accurately presented and does it cite the current literature?

Yes

If applicable, is the statistical analysis and its interpretation appropriate?

Yes

Are all the source data underlying the results available to ensure full reproducibility?

Partly

Is the study design appropriate and is the work technically sound?

Yes

Are the conclusions drawn adequately supported by the results?

Yes

Are sufficient details of methods and analysis provided to allow replication by others?

Partly

Reviewer Expertise:

Public Health, Infectious Diseases, Key Populations, Violence, Prevention, Community Centric Research

I confirm that I have read this submission and believe that I have an appropriate level of expertise to confirm that it is of an acceptable scientific standard.

# Response

**Published:** 2023-08-13

**Authors:** Janet Musimbi

**Affiliations:** Partners for Health and Development in Africa, Kenya

Dear Ms. Minja,

 Thanks a lot for reviewing the manuscript “Programmatic mapping and estimating the population size of female sex workers, men who have sex with men, people who inject drugs and transgender populations in Kenya.”

 We would like to acknowledge and thank you for the positive reviews and comments. We have had discussion with our authoring team and have revised the manuscript based on the changes proposed. Please find enclosed our response to the reviewer’s comments.

 Thanks a lot again and we remain with our kindest regards

 JANET.

**COMMENTS AND** **RESPONSES:**

**Abstract**:

 In the concluding part of the abstract there is mention that the study provided information on young KP the abstract also mentions Female PWID – did the authors intend to highlight ‘young’ key pop and specifically Female PWID despite this not being the main focus of the manuscript?

*We wanted to show that other than giving estimates of the KP in Kenya, this paper also shares data on young KP across all the typologies and specifically for the PWID, we were also able to disaggregate between male and female. We believe that providing this information will be useful for government and donors as they could look at these numbers and plan for services for these sub-populations*

**Introduction:**

 In the first part of the introduction the authors outline 3 points for why programmatic mapping and size estimations are important: “helps to estimate population size…” What are the other benefits of mapping and size estimations for the programmes apart from retention?

*Mapping and size estimations is also important as it provides evidence to decision makers on numbers within the counties and therefore supports in resource mobilization for interventions for KPs. It also shows where KPs congregate and enables implementing partners and peer educators plan for outreach. It also provides denominators which can be used to track the progress of a program and interventions*

 As this is the first time that a size estimation on the transgender population is being done, some additional information in the introduction would be beneficial.

*This is noted and have revised accordingly*

 What type of interventions (public health, clinical?) were scaled up in the 34/47 counties post the 2012 mapping and size estimation.

*The 2012 mapping and size estimations was done in the then 8 provinces of Kenya and through this results, Biomedical, Structural and behavioral were scaled up to 34 counties out of the 47. For example counties that only had programs for the FSW subpopulation, scaled up services to include services for MSM. This information has now been added to the manuscript.*

  Provide some additional information on why mapping and size estimations were only done in the 34 counties where KP programmes were being implemented. Is it cause there are no programmes in the other counties and if so some insight into why that is the case. Does it have anything to do with the previous mapping following which interventions were implemented in the 34 counties. Is the idea to update size and understand changes in the initial 34 sites only or potentially also expand. 

*This was not a stand alone research study, and was conducted as part of the  key population program  of the Kenya’s National government initiative. We focused on the counties where HIV prevention programs were already providing services to key populations.. There was a consultative process during the national technical working groups for key populations and it was agreed to conduct the KPSE exercise as part of the programs and to update size estimates of key populations in these 34 counties.*

  Last section of the Introduction: mapping and size estimation are highlighted as distinctly different steps however there seems to be a sentence missing that links the two steps.

*Thanks for highlighting this. This has been relooked at and revised.*

**Methods:**

 Were all hotspots (past and new) physically validated and if so how did the teams (implementing partners) achieve that in a 3 month period especially when there were over 10 000 female SW venues? Was the mapping, validation and size estimates completed in 3 months?

*Yes the hotspots were physically validated. This was achieved through the use of mobilizers/peer educators within the counties that were trained and used in the exercise. In our guidelines, peer educators are attached to hotspots were they provide services and meet with their peers. This meant that the spots were known and it was easy to quickly validate them as well as any new spots that were identified during the process.*

  Define operational typologies – does this refer to venue type such as bar etc?

*Yes, typology refers to the different types of hotspots and they were merged as follows: Streets, Parks/Beaches/toilets, Sex dens/brothels, residential, strip clubs/massage parlor/salon, bar with lodging, bar without lodging, guest house, uninhabited building, drug dens, casino/club and others.*

  Need clarity regarding programme selection – of the 34 counties only those that provided services to specific KPs were selected. For example, of the 34 counties, 15 provided services for PWID and hence only 15 were included?

*Similarly to the response above, we were working with Implementing partners within the counties. At the time of the size estimations only 34 counties had FSW partners implementing for the FSW, 15 counties had PWID implementing partners and 30 counties had MSM implementing partners. These are the counties that were then the focus of the size estimations as partners were already providing different interventions for the KPs in these counties. We have added this explanation in the manuscript*

  The authors mention that estimates were adjusted to account for the overlap of KPs visiting more than one spot. How did authors account for duplicate counting/estimations such that female SWs provide one estimate and PWID provide another estimate but the groups might overlap? 

*The adjustment were made independent of overlapping group participation. We agree that there are some overlapping behaviors, but we included the KP member in the group where he/she was originally counted.*

  In level 2: how were the 3-5 KP members that partook in FGDs identified?

*The KP members were identified randomly from the hotspots at the time of the size estimation hence they were found physically at the hotspot during the exerc*ise.

 Were the 3-5 members all part of the same KP group and were the KPs interviewed at the same time or separately?

*The 3-5 members were homogeneous in the sense that they belonged to the same KP group. The interview was conducted as a group so the 3-5 members participated in the same group discussion and the moderator took them through the questions , asking them and providing responses as a group.*

  Who identified the gatekeeper and how were gatekeepers approached to ensure buy in?

*The gate keepers were identified consultatively by the research teams and the KPs (social mobilizers) who were able to identify who the gatekeepers in their hotspots were to get their buy in before the size estimation exercise started at the hotspot.*

  Was any method used to guide KP member estimates?

*The members of the group discussed among themselves and after building consensus, the number which was agreed upon was used as the estimate for low, mid and high estimate. We did not use another size estimation technique.*

 For data that was being captured via paper form, what was the time frame for electronic data capture?

*The electronic format- ODK was used to upload data and submit. For instances where that could not be done onsite, then interviewer uploaded the earliest he/she accessed internet.*

  Why was the MSM data revalidated in 10 counties?

*While finalizing the estimates, the research and the program team felt that the numbers of MSM need to be revalidated as some of the numbers were different from what the program generated estimates were. Thus 5%–10% of the hotspots were randomly picked within randomly selected counties and were revalidated to check for under-reporting or over-reporting.*

 Was only verbal consent obtained and if so explain why this was sufficient if participants were debriefed and referred following participation? Also, why were participants debriefed and around what? Is the referral to care facilities standard of care for all KPs or was it specific to only those that took part in the study?

*Verbal consent was obtained and it was sufficient as data collected was aggregate in nature. No specific information about individuals* *was collected. The debriefing of participants was to share on the importance of the information shared, share on the IPs present within their areas of operations and where they could access services if they were in need of them. The referrals to care facilities is a standard care for all KPs and not only those who participated.*

  Did FGDs participants provide a percentage (eg. 9%) of KPs who were younger than 18 years or did they give an estimated value?

*They provided an estimated value which was used to compute the proportions. We also cross-checked this information using program data.*

**Results and Discussions:**

 Table 2 is divided into two parts with one part providing values while the second part provides percentages. How were the percentages calculated for hours and days of operation. Explain why hours and days of operation were not applicable for transgender people.

*We collected information the from each key population member interviewed about what re the peak days and time of their operation. This information was collected as a multiple response variable and each KP member provided information about what are the peak days and times of their operation. The proportion for a particular hour and day was calculated based on the total times and days.*

*Thanks for highlighting the missing information for Transgender populations. We cannot say that the peak day and time concept is not applicable for transgender people, as it was not collected from them. This could be one of the limitations and we have now mentioned this in the limitations.*

 How many of the KP venues overlapped? When aggregated by KP type there were a total of 14760, it was noted in the paper that key pops such as transgender people shared venues/hotspots with female SW and MSM.

*For the exercise we did not analyse which KP venues overlapped. It was however noted that transgender people share spots with FSW and MSM in 1,218 spots*.

  Under methods it is described that 30 counties were selected for mapping transgender people however the results report on information from 32 counties. Explain the inclusion of the additional counties.

*This has been relooked at and revised to 30. Thank you.*

 Include percentages in table 3 for ease of reading.

*It would be difficult to include percentages as the table provides data on the estimates per county and provides the reader with the minimum and maximum range for the estimates. If we add percentages also, the table will become very heavy.*

  Why is there no map of the transgender population?

*This has been revised and the map for the transgender population included. Thank you.*

  In the discussion the authors highlight that a high number of MSM also work as male SW. Is there any data from the mapping process to show this.

*Yes. A question that was asked and analyzed to provide this information was; Among the MSM visiting this hotspot how many sell sex for money? This information was used to develop the estimates of Male sex workers*

  Explain the significance of finding females and males who inject drugs sharing venues? 

*The significance of this was that interventions for PWID need to be tailored to ensure that the needs of the female PWIDs were taken into account and that they should not be standard. The PWID program had challenges in getting female PWIDs to come in for services and since they do share the same hotspots, it was imperative to design our programs to ensure our services appeal to them such as having female PWID only days/times for services, inclusion of care packages etc.*

  In the discussion, bring in the other counties for which there are no mapping and size estimations – is there any potential to gather data from these places.

*Yes the subsequent size estimations that was done for the country estimated for all the counties in Kenya. This was followed up later in another mapping study which included those counties as well*

  The authors cannot say that 18 counties in the whole of Kenya, rather it’s 18 counties of all those that have been mapped.

*This is noted and have been revised accordingly*

  If the authors mapped 15 counties for PWID but only account for 85% of the cohort please explain the difference?

*PWIDs are concentrated in certain parts of the country. For example, you will find more PWIDs in the coastal region, in Nairobi, parts of Central Kenya and Western County of Kisumu. Since the study was part of the prevention programs already implemented, there was no available evidence that PWIDs are available in other counties in epidemiologic proportions and were hence not mapped.*  

  How did the KP members (peer educators and outreach workers) use the information to provide services and what services?

*The data gathered had information about the names of the hotspots, the estimated number of KPs, the peak days and hours. This information was useful in planning for outreaches and scale up of services.*

  What was the total number of respondents that the estimates are based on?

 T *his would be the number of L1 and L2 interviews*

# Reviewer report: Reviewer response for version 1

**DOI:** 10.21956/gatesopenres.14899.r33183

**Published:** 2023-06-02

**Authors:** Denton Callander, Jonathan Odingo

**Affiliations:** 1 Medecins Sans Frontieres, Mombasa, Kenya; 2 The Kirby Institute, University of New South Wales, Sydney, New South Wales, Australia

The authors present an interesting study of population size estimation among ‘key populations’ in Kenya. This work is important and very difficult, so I wish to offer my congratulations on the study.

 I know this work was done several years ago and was already published as a report, so my comments are mainly focused on opportunities for further explanation and contextualisation. Some things the authors may wish to consider:

  

1. In my opinion, far greater attention should be paid to the exclusion of counties. Why was this exercise limited only to “counties where HIV prevention programs and services were implemented”? Also, were there significant differences between included/excluded counties (e.g., population size and characteristics)? Some post-hoc tests here would help.
2. The conceptual treatment of the transgender population needs some work. Notably, I did not understand why the results are not disaggregated between trans men and trans women. Perhaps this is not possible, but it needs to be explained *why *as trans people are not a homogenous population. Further, the denominator in Table 1 (“No. of transgender people per 1000 adult men”) is inappropriate, as this assumes only men can be transgender and seems to – from my reading – reflect a long history of focusing on trans women to the exclusion of their trans brothers.
3. The conceptual treatment of ‘key populations’ is not always clear. How does the counting exercise, for example, deal with the potential reality of a lesbian transgender woman who sells sex and uses drugs? The point is that these are not mutually exclusive groups, but the way they are presented here (conceptually and methodologically) seems to suggest they are.
4. I was not at all convinced by a claim in the limitations section that these results could be “an overestimation”. I encourage the authors to engage more critically with the almost certain *underestimation *of their findings: the methods demand a certain level of visibility and public engagement, which many key populations do not have as a matter of protecting their safety. As a reader, I was expecting far more contextualization of the likely underestimation as a way to draw attention to the inherent limitations of this work (and, indeed, all population mapping among vulnerable and oppressed groups). I would strongly consider beefing this up in the Discussion section.
5. A small point, but I found offensive the repeated idea of social gathering as a way for key populations to find sexual partners. I think what the authors really mean is a measure of community connection, no? It is stigmatizing to imagine that the only reason key populations might gather together is for sexual purposes; perhaps the language throughout can better reflect the complex and diverse dynamics of community and social networks.
6. I was somewhat confused by this claim on pg 7: “It was noted that people who use drugs and not necessarily injecting drugs use the same venues as those who injected drugs”. Who noted this? How was it noted? I ask because this claim is not borne out by any of the literature on drug use patterns. Injecting drugs are a very particular behaviour, which is often totally different from people who use non-injecting drugs. If this kind of claim is to be made and believed, I would definitely need more information on its source and voracity – especially because it contravenes a lot of available evidence.
7. It looks like the frequency for female sex workers in Bungoma (Table 3) is incorrectly reported as 900, which falls outside the range.
8. I invite the authors to say more about the use of “mixed methods”. What exactly was mixed about these methods (i.e., model of mixing used)? Perhaps I have missed something, but from my reading of this paper it actually is not a mixed methods design. More information may help the reader better understand if and how methods were mixed.
9. I was surprised that the authors did not contextualize their work nationally, regionally, or jurisdictionally. How do these results compare with other exercises (outside of this NASCOP one)? More references to other attempts to estimate population size would help the reader feel more confident in the results.

Is the work clearly and accurately presented and does it cite the current literature?

Partly

If applicable, is the statistical analysis and its interpretation appropriate?

Yes

Are all the source data underlying the results available to ensure full reproducibility?

Partly

Is the study design appropriate and is the work technically sound?

Yes

Are the conclusions drawn adequately supported by the results?

Partly

Are sufficient details of methods and analysis provided to allow replication by others?

Partly

Reviewer Expertise:

Population demography; public health; key populations

We confirm that we have read this submission and believe that we have an appropriate level of expertise to confirm that it is of an acceptable scientific standard, however we have significant reservations, as outlined above.

# Response

**Published:** 2023-08-13

**Authors:** Janet Musimbi

**Affiliations:** Partners for Health and Development in Africa, Kenya

Dear Mr. Jonathan,

 Thanks a lot for reviewing the manuscript “Programmatic mapping and estimating the population size of female sex workers, men who have sex with men, people who inject drugs and transgender populations in Kenya.”

 We would like to acknowledge and thank you for the positive reviews and comments. We have had discussion with our authoring team and have revised the manuscript based on the changes proposed. Please find enclosed our response to the reviewer’s comments.

 Thanks a lot again and we remain with our kindest regards.

 JANET.

**COMMENTS AND** **RESPONSE:**

 1.

 In my opinion, far greater attention should be paid to the exclusion of counties. Why was this exercise limited only to “counties where HIV prevention programs and services were implemented”? Also, were there significant differences between included/excluded counties (e.g., population size and characteristics)? Some post-hoc tests here would help.

*Thanks a lot for the comment. This was not a stand-alone research study and was conducted as part of the key population program of the Kenya’s National government initiative. We focused on the counties where HIV prevention programs are already providing services to key populations. There was a consultative process during the national technical working groups for key populations and it was agreed to conduct the KPSE exercise as part of the programs and to update size estimates of key populations in these counties. We focused on 34 counties out of the total 47 counties in Kenya, where KP programs are providing services.*

 2.

 The conceptual treatment of the transgender population needs some work. Notably, I did not understand why the results are not disaggregated between trans men and trans women. Perhaps this is not possible, but it needs to be explained *why *as trans people are not a homogenous population. Further, the denominator in Table 1 (“No. of transgender people per 1000 adult men”) is inappropriate, as this assumes only men can be transgender and seems to – from my reading – reflect a long history of focusing on trans women to the exclusion of their trans brothers.

*This study did not include transmen, and only focused on transwomen, hence the denominator is 1000 adult men.  For this mapping exercise we used the broader term of transgender, as been used in other similar studies. As per your suggestion, we have now indicated this in the manuscript, but would like to continue using the term Transgender.*

 3.

 The conceptual treatment of ‘key populations’ is not always clear. How does the counting exercise, for example, deal with the potential reality of a lesbian transgender woman who sells sex and uses drugs? The point is that these are not mutually exclusive groups, but the way they are presented here (conceptually and methodologically) seems to suggest they are.

*As already mentioned, this study focused on transmen and Lesbian/Transgender women were not included. When at the hotspot we only focused on the population defined. We had Social mobilizers from these hotspots involved in the exercise and we had definitions of these populations. Different levels of data collection were done and the social mobilizers had a clear instruction on which type of KPs to interview.*

 4.

 I was not at all convinced by a claim in the limitations section that these results could be “an overestimation”. I encourage the authors to engage more critically with the almost certain *underestimation *of their findings: the methods demand a certain level of visibility and public engagement, which many key populations do not have as a matter of protecting their safety. As a reader, I was expecting far more contextualization of the likely underestimation as a way to draw attention to the inherent limitations of this work (and, indeed, all population mapping among vulnerable and oppressed groups). I would strongly consider beefing this up in the Discussion section.

*Thanks for highlighting this. We agree with your comment and the word overestimation has been removed from the manuscript text.*

 5.

 A small point, but I found offensive the repeated idea of social gathering as a way for key populations to find sexual partners. I think what the authors really mean is a measure of community connection, no? It is stigmatizing to imagine that the only reason key populations might gather together is for sexual purposes; perhaps the language throughout can better reflect the complex and diverse dynamics of community and social networks.

*This programmatic mapping was conducted to identify populations that are actively seeking partners at venues (FSW/MSM and TG) and/or congregating for injecting (PWIDs). We are cognizant that this is not the only reason why KPs congregate but due to the nature of how the questions were asked, this was the key factor as to why the KPs were at the hotspots. We take into account your concern and have tried to address it.*

 6.

 I was somewhat confused by this claim on pg 7: “It was noted that people who use drugs and not necessarily injecting drugs use the same venues as those who injected drugs”. Who noted this? How was it noted? I ask because this claim is not borne out by any of the literature on drug use patterns. Injecting drugs are a very particular behaviour, which is often totally different from people who use non-injecting drugs. If this kind of claim is to be made and believed, I would definitely need more information on its source and voracity – especially because it contravenes a lot of available evidence.

*The actual field data collected shows that at various drug spots we had both injecting and non-injecting and the data has been uploaded as well. On the 402 spots identified and mapped, the study team identified 16,063 injecting and 10,951 non-injecting drug users, who were using the same hotspot. In Kenya this is a common occurrence and we had specific questions asked to differentiate between the two.*

 7.

 It looks like the frequency for female sex workers in Bungoma (Table 3) is incorrectly reported as 900, which falls outside the range.

*Thank you for this and this has been corrected to 3900.*

 8.

 I invite the authors to say more about the use of “mixed methods”. What exactly was mixed about these methods (i.e., model of mixing used)? Perhaps I have missed something, but from my reading of this paper it actually is not a mixed methods design. More information may help the reader better understand if and how methods were mixed.

*Agreed. Since this paper represents more of the programmatic data, we are removing the term mixed methods approach from the text and are saying that quantitative data was collected.*

 9.

 I was surprised that the authors did not contextualize their work nationally, regionally, or jurisdictionally. How do these results compare with other exercises (outside of this NASCOP one)? More references to other attempts to estimate population size would help the reader feel more confident in the results.

*Thanks for the suggestion. We have now added some more estimates to contextualize the work nationally and regionally.*
